# Early Discontinuation of Prophylactic Antibiotics Is Not Associated with Increased Surgical Site Infection Risk in Spine Surgery: A Nationwide Cohort Study

**DOI:** 10.3390/antibiotics15030272

**Published:** 2026-03-06

**Authors:** Sangjun Park, Jun-Seok Lee, Young-Hoon Kim, Sang-Il Kim, Youngjin Kim, Sukil Kim, Hyung-Youl Park

**Affiliations:** 1 Department of Orthopedic Surgery, Eunpyeong St. Mary’s Hospital, College of Medicine, The Catholic University of Korea, Seoul 03312, Republic of Korea; jack2020@korea.ac.kr (S.P.); junband@naver.com (J.-S.L.); 2 Department of Orthopedic Surgery, Seoul St. Mary’s Hospital, College of Medicine, The Catholic University of Korea, Seoul 06591, Republic of Korea; boscoa@catholic.ac.kr (Y.-H.K.); sang1kim@catholic.ac.kr (S.-I.K.); 3 Department of Preventive Medicine and Public Health, College of Medicine, The Catholic University of Korea, Seoul 06591, Republic of Koreasikimmd@catholic.ac.kr (S.K.)

**Keywords:** antibiotic prophylaxis, spine surgery, surgical wound infection

## Abstract

**Background/Objectives**: Surgical site infection (SSI) remains a significant complication following spine surgery, yet the optimal duration of prophylactic antibiotic administration remains debated. We investigated the association between prophylactic antibiotic duration and SSI rates following spine surgery using a nationwide claims database. **Methods**: This retrospective cohort study analyzed data from the Health Insurance Review and Assessment Service quality assessment database across four assessment waves (2014–2020, sixth to ninth). Adult patients (aged ≥19 years) undergoing elective spine surgery (decompression, instrumented fusion, vertebroplasty, or kyphoplasty) were categorized into two groups based on prophylactic antibiotic duration: <24 h or ≥24 h. Surgery type was the primary surgical categorization, while surgery site (cervical, thoracic, lumbar) was assessed separately in supplemental analyses. Primary outcomes included SSI, non-surgical-site infections, and total postoperative infections within 3 months. Multivariable logistic regression was performed to identify independent predictors of infection. **Results**: Of 82,840 patients included, 19,988 (24.1%) discontinued prophylactic antibiotics within 24 h and 62,852 (75.9%) continued antibiotics for ≥24 h. The <24 h group demonstrated significantly lower SSI rates compared to the ≥24 h group (0.16% vs. 1.47%, *p* < 0.05). After adjustment for confounders, prolonged antibiotic prophylaxis (≥24 h) was associated with increased odds of SSI (adjusted odds ratio [aOR] = 10.73, 95% CI = 7.30–15.79), non-surgical-site infections (aOR = 16.06, 95% CI = 13.11–19.67), and total postoperative infections (aOR = 17.82, 95% CI = 14.83–21.42). **Conclusions**: In this nationwide cohort, early discontinuation of prophylactic antibiotics within 24 h was not associated with increased SSI risk. Prolonged antibiotic prophylaxis beyond 24 h was associated with higher SSI rates, although confounding by indication likely contributed to this finding. These results are consistent with current guideline recommendations for limiting prophylactic antibiotic duration to 24 h or less in routine spine surgery, while recognizing that individualized approaches may be warranted in some high-risk patients.

## 1. Introduction

Surgical site infection (SSI) remains one of the most consequential complications following spine surgery, significantly impacting patient outcomes and resource burden. The incidence of SSI in spine surgery ranges from 0.7% to 12% depending on the complexity of the procedure, with instrumented fusion procedures carrying higher infection rates than simple decompression surgery [1,2]. Deep SSI following spine surgery is particularly devastating, with incidence of 1.5%, with 25% of patients suffering from persistent infection that requires repeat surgical debridement [3]. Patients who develop postoperative spinal infections demonstrate significantly higher one-year mortality rates compared to those without infection [4]. The economic burden, being equally profound, adds up to nearly $93,741 in direct costs per episode for deep SSI patients, contributing to an estimated annual national expenditure exceeding a total of $900 million for spinal SSI management alone [5,6].

Although administration of perioperative antibiotics for prophylactic measures is a cornerstone of SSI prevention [7,8,9], emerging evidence also suggests that indiscriminate prolonged application of antibiotics not only fails to reduce SSI rates but may paradoxically be associated with adverse outcomes. The landmark study by Harbarth et al. demonstrated that continuing antibiotic prophylaxis beyond 48 h after cardiac surgery was ineffective in reducing SSI and was associated with a 60% increased risk of acquired antimicrobial resistance [10]. In spine surgery specifically, Porter et al. reported that antimicrobial prophylaxis over three or more days was associated with 1.8-fold increased odds of prolonged hospitalization and 3.5-fold increased odds of 30-day reoperation [11]. Additionally, a recent meta-analysis confirmed that extended postoperative antibiotic prophylaxis in adult spine surgery demonstrates no significant value in reducing SSI rates while being associated with increased length of hospital stay and higher costs [12,13].

Notably, the guideline recommendations for prophylactic antibiotic duration have become progressively more restrictive over the study period. The 2013 ASHP/IDSA/SIS/SHEA guidelines recommended prophylaxis duration of less than 24 h for most surgical procedures, including spine surgery, and stated that the presence of surgical drains does not justify extended administration [8]. In 2017, the CDC guideline represented a significant further restriction, recommending that additional prophylactic antibiotics should not be administered after surgical incision closure, even in the presence of a drain [14]. The 2022 SHEA/IDSA update reinforced this position, recommending discontinuation of prophylaxis after skin closure for clean and clean-contaminated procedures [15]. In South Korea, the Health Insurance Review and Assessment Service (HIRA) quality assessment (QA) program implemented corresponding changes: the earlier QA waves (sixth through eighth, 2014–2017) evaluated antibiotic duration without strict temporal limitations, while the ninth QA wave (2020) imposed a mandatory 24 h postoperative restriction for all elective spinal surgeries, which likely contributed to the higher proportion of early discontinuation observed during this period. These evolving guidelines provide important context for interpreting temporal trends in the present study.

Although there is mounting evidence questioning the benefit of prolonged antibiotic prophylaxis, large-scale population-based studies examining this relationship in spine surgery remain limited, particularly in Asian populations. The purpose of this study was to investigate the association between the duration of prophylactic antibiotic administration and SSI rates following spine surgery using a nationwide claims database. We hypothesized that early discontinuation of prophylactic antibiotics within 24 h would not be associated with increased SSI rates compared to prolonged prophylaxis beyond 24 h.

## 2. Results

### 2.1. Baseline Demographics by Duration of Antibiotic Usage

**Figure 1:**
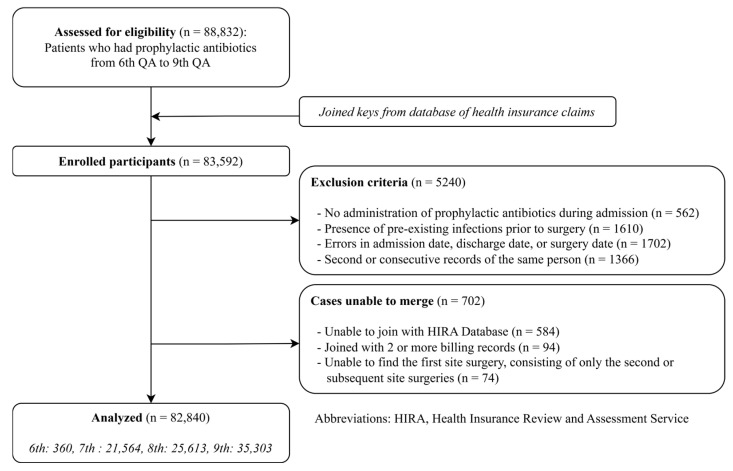
Flow chart of study participants.

A total of 82,840 patients were enrolled in the study after applying inclusion and exclusion criteria (Figure 1). Of these, 19,988 patients (24.13%) were administered prophylactic antibiotics for <24 h after surgery, while 62,852 patients (75.87%) received prophylactic antibiotics for ≥24 h after surgery. Baseline characteristics of the study population are presented in Table 1. Patients in the <24 h discontinuation group were significantly older (68.33 ± 14.96 years vs. 62.99 ± 15.03 years, *p* < 0.05) and more likely to be female (64.30% vs. 54.49%, *p* < 0.05) compared to the ≥24 h group. The proportion of patients treated in the ninth QA period was higher in the <24 h group (61.11% vs. 36.73%, *p* < 0.05), reflecting stricter QA standards implemented during this period. Additional analyses performed with the patient cohort limited to decompression and instrumented fusion surgeries showed similar patterns and are presented in Supplemental Table S1.

Supplemental Figure S1 shows the changes in the prevalence of each assessment item across the sixth, seventh, eighth, and ninth QA periods. There was a statistically significant decrease in mean antibiotic duration over time (*p* = 0.046), reflecting improved adherence to guideline-recommended practices following the implementation of stricter QA criteria in the ninth wave.

### 2.2. Differences in SSI Rates by Duration of Antibiotic Usage

Table 2 describes the postoperative infection differences between the <24 h and ≥24 h discontinuation groups. The <24 h discontinuation group had significantly lower rates of SSIs (31 cases, 0.16% vs. 922 cases, 1.47%, *p* < 0.05), non-surgical-site infections (122 cases, 0.61% vs. 2817 cases, 4.48%, *p* < 0.05), and total postoperative infections (153 cases, 0.77% vs. 3652 cases, 5.81%, *p* < 0.05). When examining specific types of SSI, the <24 h group consistently showed lower rates across all categories: pus or purulent drainage from incision sites or organs (0.04% vs. 0.22%, *p* < 0.05), positive culture results from incision sites or organs (0.01% vs. 0.14%, *p* < 0.05), surgical wounds that ruptured spontaneously or were opened by a surgeon with signs of infection (0.03% vs. 0.19%, *p* < 0.05), evidence of abscess or infection in deep tissue or organs by histopathological or radiological examination (0.00% vs. 0.03%, *p* < 0.05), and diagnosis of SSI by a surgeon, attending physician, or infectious disease specialist (0.09% vs. 0.99%, *p* < 0.05). Subgroup analysis in patients who underwent only decompression and instrumented fusion showed consistent results (Supplemental Table S2).

### 2.3. Multivariable Logistic Regression Analysis for SSI

**Figure 2:**
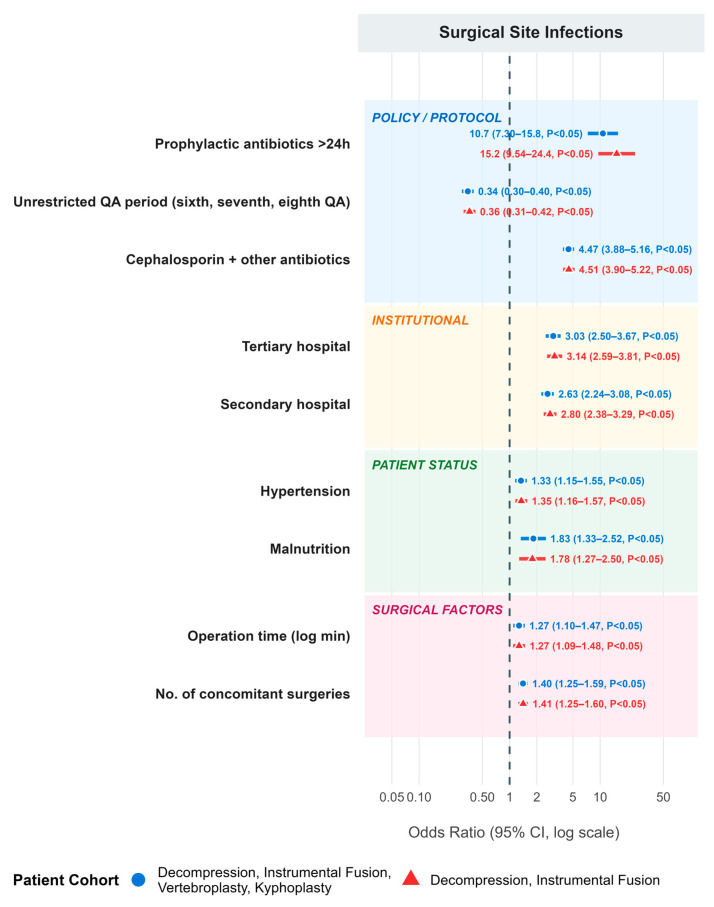
Forest plot of predicting factors regarding multivariable logistic regression analysis for surgical site infection.

Results from multivariable logistic regression are shown in Table 3. Compared to the <24 h discontinuation group (reference), the ≥24 h discontinuation group was associated with significantly increased odds of SSIs (aOR = 10.732, 95% CI = 7.296–15.785, *p* < 0.05), non-surgical-site infections (aOR = 16.062, 95% CI = 13.114–19.671, *p* < 0.05), and total postoperative infections (aOR = 17.823, 95% CI = 14.828–21.422, *p* < 0.05). The ninth QA period (vs. earlier periods) was associated with significantly reduced odds of SSIs (aOR = 0.345, 95% CI = 0.297–0.401), reflecting improved infection control practices and stricter prophylactic antibiotic protocols. A summary of the results is illustrated as a forest plot in Figure 2.

### 2.4. Subgroup Analysis with Patients Who Underwent Only Decompression and Instrumented Fusion

Subgroup analysis in patients with only decompression and instrumented fusion showed similar but more pronounced associations (Supplemental Table S3). In this subgroup, prolonged antibiotic administration was associated with even higher odds of SSIs (aOR = 15.250, 95% CI = 9.538–24.383), non-surgical-site infections (aOR = 31.439, 95% CI = 23.888–41.377), and total postoperative infections (aOR = 33.677, 95% CI = 26.439–42.896). Supplemental Table S4 shows the number of patients with SSIs by surgery type and location. Regardless of surgery type, the <24 h group showed lower SSI rates than the ≥24 h group in cervical and thoracic locations. In lumbar surgery, which constituted the majority of procedures, the pattern was consistent with the overall findings.

## 3. Discussion

Our study examined the association between prophylactic antibiotic duration and surgical site infection rates following spine surgery using a nationwide insurance claims database. Principal findings demonstrated that patients who received prophylactic antibiotics for 24 h or longer had significantly higher SSI rates compared to those receiving them for less than 24 h, with an adjusted odds ratio of 10.732. However, these findings should be interpreted in the context of an observational study design and the strong potential for confounding by indication, as discussed below. Rather than suggesting a causal detrimental effect of prolonged prophylaxis, our results only show consistency with selective prolongation of antibiotics in patients with higher baseline surgical complexity and perceived infection risk [16,17,18,19,20].

Our findings agree with multiple studies across various surgical specialties demonstrating the lack of benefit from prolonged antibiotic prophylaxis. A randomized controlled trial (RCT) by Takemoto et al. involving 314 patients undergoing multilevel thoracolumbar spine surgery found no difference in SSI rates between 24 h prophylaxis and drain-duration antibiotics (12.4% vs. 13.2%; *p* = 0.48), concluding that the presence of surgical drains does not justify extended antibiotic administration [21]. Similarly, Nagata et al. conducted a multicenter cluster randomized noninferiority trial of 1211 patients and demonstrated that prophylaxis of less than 24 h was noninferior to 24–48 h, with numerically lower infection rates in the shorter-duration group [22]. Pivazyan et al. conducted a meta-analysis analyzing 2446 spine surgery patients and found no reduction in deep SSI with prolonged prophylaxis (OR = 1.10, 95% CI = 0.69–1.74), while observing a concerning trend toward increased multidrug-resistant organisms in the prolonged prophylaxis group [23]. Perhaps most definitively, the WHO-endorsed meta-analysis by de Jonge et al. encompassing 52 RCTs and 19,273 participants concluded that postoperative continuation of antibiotics provided no benefit when best practices were followed (RR = 1.04, 95% CI = 0.85–1.27), stating that prolonged prophylaxis “unnecessarily exposes patients to risks” [24].

While the mechanisms linking prolonged antibiotics to increased surgical site infection (SSI) rates remain unclear, microbiology offers clues. One hypothesis suggests that microbiome pathogens hide inside immune cells, traveling to surgical wounds to release infections. Thus, antibiotic-induced dysbiosis creates an endogenous SSI reservoir immune to standard prophylaxis [25,26]. Furthermore, extended antibiotics disrupt healthy gut microbiota, enabling pathogen colonization [27,28]. This aligns with findings that most healthcare-associated infections are inherently endogenous [29] and resistant to the administered antibiotics [30,31]. Consequently, joint guidelines (ASHP, IDSA, SIS, SHEA) recommend limiting prophylaxis to under 24 h, as longer durations provide no added SSI prevention but increase risks of microbiological resistance and drug-related adverse events [8].

The adverse consequences of prolonged antibiotic prophylaxis extend beyond SSI to include dose-dependent increases in acute kidney injury and *C. difficile* infection [32]. A study of a multicenter national cohort of veterans revealed that across all major surgical procedures, longer duration of administering prophylactic antibiotics resulted not in SSI rate decrease, but rather in increases in harmful adverse effects [32]. It has been shown that it also affects various in-hospital outcomes such as longer length of stay and higher chance of reoperation specifically in lumbar spine surgery [11]. These findings emphasize that prolonged prophylaxis carries quantifiable harm that must be weighed against its purported benefits.

Tertiary hospitals were independently associated with higher SSI odds (aOR = 3.029), consistent with patterns observed in analyses of the same database [33]. At first glance, this may seem unintuitive, since tertiary hospitals are known to employ rigorous infection control standards. However, we believe that this finding is actually reflecting a referral bias: tertiary centers manage the most complex surgical cases, including multilevel instrumented procedures and patients with significant comorbidities, which simultaneously increase both SSI risk and thus the likelihood that clinicians would prolong antibiotic prophylaxis. The ninth QA period (2020) was also independently associated with lower SSI risk (aOR = 0.345), likely reflecting the stricter policy applied to the usage of prophylactic antibiotics. Since early antibiotic discontinuation was more common during the ninth QA wave (61.1% of the <24 h group), the protective association of shorter prophylaxis may be partly attributable to these concurrent improvements in perioperative care, despite adjustment for QA period in the regression model.

The SSI rate of 0.16% in the <24 h group is lower than rates typically reported in clinical surveillance studies of spine surgery (0.7–12%) [1,2]. We suspect that this discrepancy stems from multiple causes, one being that patients who underwent vertebroplasty or kyphoplasty were all included in our study, which diluted the original SSI rate since such procedures result in very low SSI rates. That is, the high proportion of minimally invasive vertebroplasty and kyphoplasty procedures in the <24 h group could have contributed to the low observed rate. This outcome misclassification, if non-differential between groups, would actually bias results toward the null, suggesting that the observed differences should be interpreted cautiously. This is not only because of the type of procedures; we also believe that only cases of infection recorded during the study period being considered had a lowering effect on the overall SSI rate.

This study has several limitations. The HIRA QA database, although it allows a large-scale analysis, lacks clinical details such as surgical invasiveness scores, wound classification, body mass index, American Society of Anesthesiologists (ASA) physical status classification, laboratory values, and microbiological culture results that would enhance risk adjustment [33]. The HIRA database does not contain body mass index, body weight, or other anthropometric data [34]. Obesity is a well-established independent risk factor for SSI in spine surgery, with meta-analytic evidence demonstrating that each 5-unit increase in BMI raises spinal SSI risk by approximately 21% [35,36]. Furthermore, obesity may influence antibiotic dosing and tissue penetration of prophylactic cefazolin [37,38]. The inability to adjust for this variable represents a notable limitation. We were not able to ascertain the specific antibiotic agents used, dosing regimens, or redosing practices. In addition, temporal variances such as demographic differences between hospitals and QA waves could have influenced the outcomes through potential bias. While the hospitals’ alignment with current guideline recommendations provided some amount of integrity to the findings, our study may not have captured the nuanced differences in antibiotic exposure. Because no standardized patient-level protocol dictated the decision to continue or discontinue antibiotics at 24 h, the observed grouping reflects a combination of institutional practices, evolving QA incentives, and individual surgeon judgment, further reinforcing the potential for confounding by indication. Lastly, we could not assess the appropriateness of antibiotic usage in each patient or each hospital, since no information was given on the type or class of the confirmed pathogens.

A critical interpretive consideration is confounding by indication, whereby clinicians may have selectively prolonged antibiotic prophylaxis in patients perceived to be at higher baseline infection risk [16,17,18,39]. The significantly longer operation times, higher proportions of decompression and instrumented fusion, and greater comorbidity burden observed in the ≥24 h group support this possibility. Notably, although we adjusted for operation time in our multivariable model, the length of surgery may represent more than a simple confounding variable; it can directly influence the decision to assign patients to extended antibiotic prophylaxis, effectively functioning as a determinant of group allocation rather than merely an independent risk factor. While our multivariable model adjusted for available confounders including surgery type, operation time, hospital type, comorbidities, and QA period, unmeasured factors such as body mass index, ASA classification, wound class, and surgeon-level clinical judgment could not be captured in the HIRA claims database. Accordingly, the large, adjusted odds ratio of 10.73 should be interpreted cautiously as reflecting both the true association and residual confounding. The observed pattern, in which the group receiving longer antibiotic courses paradoxically demonstrated higher SSI rates, is consistent with the confounding-by-indication signature described in prior observational antibiotic studies [40]. We emphasize that our findings represent associations and should not be interpreted as evidence that prolonged prophylaxis causally increases infection risk.

Reverse causality is also a plausible explanation. Patients who developed early postoperative complications or clinical signs suggestive of infection may have had their antibiotic courses extended beyond 24 h as a reactive, therapeutic measure rather than as part of a prospectively planned prophylactic regimen. Because the HIRA database records total antibiotic duration without distinguishing between prophylactic and therapeutic intent, this temporal ambiguity cannot be resolved in the present study. Additionally, longer operation times and hospitalization in the ≥24 h group may reflect greater surgical invasiveness that independently increases both infection risk and the likelihood of prolonged antibiotic administration. Furthermore, subclinical preoperative colonization or infection that did not meet the exclusion threshold may have gone undetected, potentially contributing to both the decision to extend antibiotic prophylaxis and the subsequent development of SSI.

The heterogeneous distribution of SSI across spinal regions in our study warrants consideration. Published data show that regional SSI variation can be heavily influenced by surgical approach and microbial ecology rather than anatomical location alone. Zhou et al. found that posterior approaches carried SSI rates approximately twice as high as anterior approaches [1], and Pralea et al. demonstrated that Gram-negative pathogens occur predominantly in lumbar procedures, attributable to contamination from perianal and genitourinary flora [41]. Cervical surgery is frequently performed via an anterior approach with smaller incisions and less muscle dissection, which generally results in lower SSI rates. Our findings differ from those of Alfin et al., who reported higher lumbar SSI rates in a single developing-country neurosurgical center [42]; this discrepancy likely reflects differences in surgical case mix, infrastructure, perioperative protocols, and healthcare system settings between a nationwide Korean cohort and a single tertiary center in a developing country. To address the imbalance in surgery type distribution between groups, we performed a prespecified subgroup analysis excluding vertebroplasty and kyphoplasty, which confirmed that the association persisted in a more homogeneous surgical population (aOR = 15.250 for SSI among decompression and instrumented fusion patients only). Also, as there are only a few cases of SSI, we utilized multivariable logistic regression analysis rather than propensity score-based matching/weighing methods. Given the rarity of SSI events, propensity score matching could have substantially reduced the number of infection cases and limited statistical power [43,44].

Despite these limitations, however, our analysis of 82,840 patients across four national QA waves provides population-level evidence that routine extension of prophylactic antibiotics beyond 24 h was not associated with lower SSI rates in elective spine surgery. While causal inference cannot be established from this observational design, the consistency of our findings with prior RCTs and systematic reviews [12,21,22,23] supports current guideline recommendations for limiting prophylactic antibiotic duration. Future prospective studies with detailed clinical data, including patient risk stratification and microbiological outcomes, are warranted to further clarify the optimal prophylactic antibiotic strategy across different risk profiles in spine surgery.

## 4. Materials and Methods

### 4.1. Database

This study utilized the Health Insurance Review and Assessment Service (HIRA) quality assessment (QA) database, which contains comprehensive data from all healthcare institutions in South Korea that participated in the nationwide prophylactic antibiotic QA program [45]. HIRA conducted nine consecutive QA waves from 2007 to 2020, focusing on prophylactic antibiotic use in surgical interventions, including spinal surgeries. Since the 1st through 5th QA waves (2007–2012) focused on other surgical categories and did not include spinal procedures, our study utilized data from the sixth through ninth QA waves (2014–2020), which represent all four assessment periods during which the QA program specifically evaluated prophylactic antibiotic use in elective spinal surgeries. A key strength of the QA database is its rigorous and structured evaluation process, which includes detailed documentation of prophylactic antibiotic use, patient characteristics, surgical complexity, and postoperative outcomes including SSIs. SSIs are systematically identified and recorded based on predefined diagnostic criteria, ensuring consistency and accuracy across QA waves. The QA database was linked to the national health insurance claims database using anonymous join keys to incorporate additional information on patient comorbidities and medical history that were not captured within the QA dataset alone. This integration enabled a comprehensive assessment of patient risk factors while maintaining data standardization.

#### Patient and Public Involvement

This study was approved by HIRA (approval no. M20230221003) and by the Institutional Review Board of Eunpyeong St. Mary’s Hospital, Catholic University of Korea (approval no. PC23ZISI0031). The requirement for informed consent was waived due to the retrospective design and use of de-identified data.

### 4.2. Study Design

We conducted a retrospective cohort study analyzing data from four QA waves: the sixth QA (January to March 2014), seventh QA (September to November 2015), eighth QA (October to December 2017), and ninth QA (October to December 2020). All patients aged 19 years or older who underwent elective spine surgery during the QA periods were eligible for inclusion. Patients who underwent spine surgery including decompression, instrumented fusion, vertebroplasty, and kyphoplasty were identified using predefined procedural codes aligned with the original QA criteria. Patients were excluded if they had (1) pre-existing infections prior to surgery, defined as either prior antibiotic use for a confirmed infection or physician documentation of a condition necessitating antibiotic treatment before surgery; (2) no administration of prophylactic antibiotics during admission; (3) any residing errors in admission, discharge, or surgery dates; (4) multiple surgical procedures within a single admission or concurrent spinal and non-spinal operations; or (5) two or more consecutive records of the same person. Patients were categorized into two groups based on the duration of prophylactic antibiotic administration: the <24 h discontinuation group, in which prophylactic antibiotics were discontinued within 24 h after surgery, and the ≥24 h discontinuation group, in which prophylactic antibiotics were continued for more than 24 h after surgery.

#### 4.2.1. Assessment Criteria of QA

The QA criteria for prophylactic antibiotics in spinal surgeries changed across the QA waves. In the sixth, seventh, and eighth QA waves, the guidelines maintained a relatively flexible approach to antibiotic selection while restricting the use of aminoglycosides, third-generation cephalosporins, and combination therapies. The overall duration of antibiotic use was evaluated, but no strict temporal limitation was imposed. The ninth QA wave (October to December 2020) introduced substantially stricter guidelines, limiting the selection exclusively to first- or second-generation cephalosporins for all spinal surgeries (with exceptions for documented antibiotic allergies) and implementing a 24 h postoperative restriction on prophylactic antibiotic duration. Throughout all waves, the timing of the initial antibiotic administration remained consistent, requiring administration within 1 h before skin incision. No standardized, patient-level protocol dictated the decision to discontinue antibiotics at 24 h versus continuing beyond 24 h; this decision was made at the discretion of the treating physician and was influenced by institutional practices and the prevailing QA criteria.

#### 4.2.2. Outcome Definitions

The primary outcomes were (1) surgical site infections (SSIs), (2) non-surgical-site infections, and (3) total postoperative infections, all assessed within 3 months of surgery. SSIs were diagnosed according to predefined criteria consistent with the Centers for Disease Control and Prevention (CDC) definition [14], encompassing superficial incisional, deep incisional, and organ/space infections. The HIRA QA program identified SSIs using standardized clinical, microbiological, and radiological criteria, requiring the presence of at least one of the following: (a) purulent discharge from the incision site or drainage catheter; (b) positive bacterial cultures obtained under aseptic conditions from the incision site, deep tissues, or internal organs; (c) surgical wounds that dehisced spontaneously or were deliberately opened by a surgeon in the presence of one or more signs of infection, including fever exceeding 38 °C, localized pain, tenderness, or erythema; (d) abscess or other evidence of infection identified in deep tissue, organs, or body cavities through imaging or histopathological examination; or (e) clinical diagnosis of SSI by the treating surgeon, attending physician, or infectious disease specialist. SSI diagnoses were prospectively recorded by the treating physicians during each QA period, and the QA evaluation included a cross-verification process to ensure consistency between reported diagnoses and corresponding medical records [46].

### 4.3. Variables

Patient demographic and clinical characteristics were collected, including age, sex, insurance type, and hospital type (tertiary, general, or primary). Comorbidities were identified using International Classification of Diseases, tenth Revision (ICD-10) diagnostic codes from records during and prior to the index hospitalization, including diabetes mellitus (DM; E10–E14), hypertension (I10–I15), malnutrition (E40–E46), uncontrolled diabetes (E10.64, E11.64, E12.64, E13.64, E14.65), and skin or soft tissue infections (L00–L08). Surgical variables included surgery type, surgery site (cervical, thoracic, lumbar), number of concomitant spine surgeries, operation time, total hospitalization days, and types of prophylactic antibiotics used. The antibiotic category “other antibiotics only” includes cases in which agents other than first- or second-generation cephalosporins were used as the sole prophylactic agent, including patients with documented cephalosporin allergies, institutional protocols incorporating vancomycin or broader-spectrum agents, and cases from the earlier QA waves when antibiotic selection guidelines were more flexible. The “both” category includes cases receiving a first- or second-generation cephalosporin in combination with another antibiotic class, reflecting combination prophylaxis regimens used particularly in the earlier QA periods.

### 4.4. Statistical Analyses

Continuous variables were summarized as means with standard deviations (SDs) or medians with interquartile ranges (IQRs) as appropriate and compared using Student’s *t*-test or the Mann–Whitney U test. Categorical variables were expressed as frequencies and percentages and compared using Pearson’s chi-square test or Fisher’s exact test. Multivariable logistic regression analysis was performed to identify independent predictors of SSIs, non-surgical-site infections, and total postoperative infections. Variables included in the model were: antibiotic discontinuation group, age, sex, QA period (sixth–eighth vs. ninth QA), antibiotics used (first- or second-generation cephalosporin only, other antibiotics only, or both), insurance type, hospital type, surgery type, comorbidities (diabetes, hypertension, malnutrition), skin or soft tissue infection history, allergy to antibiotics, operation time (log-transformed due to exponential distribution), and number of concomitant spine surgeries. Results were presented as adjusted odds ratios (aORs) with 95% confidence intervals (CIs). Additional analyses were performed to account for the different burden of procedures, excluding vertebroplasty and kyphoplasty procedures, which are considered relatively low-burden interventions. Model diagnostics included assessment of multicollinearity using variance inflation factors (VIF < 2.5 for all covariates) and max-rescaled R^2^ values. The Hosmer–Lemeshow goodness-of-fit test was considered but interpreted cautiously, as it has excessive statistical power in large samples (N > 10,000), rejecting the null hypothesis for practically irrelevant departures from perfect fit [47]. All statistical analyses were performed using SAS software version 7.1 (SAS Institute, Cary, NC, USA) and R version 4.5.2 (R Core Team, Vienna, Austria, 2026). A two-tailed *p*-value < 0.05 was considered statistically significant.

## 5. Conclusions

In this nationwide claims-based cohort of 82,840 patients undergoing spine surgery, discontinuation of prophylactic antibiotics within 24 h was associated with lower SSI rates compared to prolonged administration beyond 24 h. However, this finding likely stems from confounding by indication, as patients receiving longer antibiotic courses had greater surgical complexity and higher baseline risk profiles. Our results suggest that routine extension of prophylactic antibiotics beyond 24 h does not demonstrate a protective association at the population level, which is consistent with current guideline recommendations. Nevertheless, these findings should not be interpreted as discouraging individualized antibiotic strategies in clearly defined high-risk patients, where clinical judgment remains essential. Antimicrobial stewardship programs should continue to target prolonged surgical prophylaxis as a key intervention for reducing unnecessary antibiotic exposure while ensuring that clinical decision-making accounts for individual patient risk profiles.

## Figures and Tables

**Table 1:** Baseline characteristics of study participants who had either decompression, instrumented fusion, vertebroplasty, or kyphoplasty ^†^.

Characteristics	Prophylactic Antibiotics Discontinued Within 24 h of Surgery	Prophylactic Antibiotics Continued for More than 24 h After Surgery	*p*-Value
	(*n* = 19,988, 24.13%)	(*n* = 62,852, 75.87%)	
Age, years	68.33 (14.96)	62.99 (15.03)	<0.05
Round			<0.05
sixth	157 (0.79%)	203 (0.32%)	
seventh	3404 (17.03%)	18,160 (28.89%)	
eighth	4212 (21.07%)	21,401 (34.05%)	
ninth	12,215 (61.11%)	23,088 (36.73%)	
Sex			<0.05
Male	7135 (35.70%)	28,602 (45.51%)	
Female	12,853 (64.30%)	34,250 (54.49%)	
Comorbidities ^a^			
DM	5810 (29.07%)	17,213 (27.39%)	<0.05
Hypertension	8498 (42.52%)	23,006 (36.60%)	<0.05
History ^a^			
Malnutrition	489 (2.45%)	1079 (1.72%)	<0.05
Uncontrolled DM	111 (0.56%)	316 (0.50%)	0.37
Skin or soft tissue infection	820 (4.10%)	2784 (4.43%)	<0.05
Insurance types			0.27
Health insurance coverage	18,827 (94.19%)	59,330 (94.40%)	
Medical aids	1161 (5.81%)	3522 (5.60%)	
Hospital types			<0.05
Tertiary	4999 (25.01%)	4618 (7.35%)	
General	5344 (26.74%)	12,542 (19.95%)	
Hospital	9645 (48.25%)	45,692 (72.70%)	
Antibiotics used			<0.05
1st- or 2nd-generation cephalosporin only	18,932 (94.72%)	51,669 (82.21%)	
Other antibiotics only	542 (2.71%)	2312 (3.68%)	
1st- or 2nd-generation cephalosporin, and other antibiotics—both	514 (2.57%)	8871 (14.11%)	
Surgery type			<0.05
Vertebroplasty and kyphoplasty	10,234 (51.20%)	13,603 (21.64%)	
Decompression	7047 (35.26%)	38,925 (61.93%)	
Instrumented fusion	2707 (13.54%)	10,324 (16.43%)	
Allergy to antibiotics, presence	322 (1.61%)	1147 (1.82%)	<0.05
Number of concomitant spine surgeries, means (SD)	1.30 (0.56)	1.29 (0.52)	<0.05
Operation times (minutes)	45.0 (20.0–101.0)	85.0 (50.0–134.0)	<0.05
Total hospitalization days (days)	6.0 (3.0–10.0)	11.0 (7.0–16.0)	<0.05

^†^ Values are either mean (SD), median (IQR), or N (%). ^a^ Positive if there was a past history before discharge.

**Table 2:** Postoperative infection differences between the <24 h discontinuation group and ≥24 h discontinuation group in patients who had either decompression, instrumented fusion, vertebroplasty, or kyphoplasty ^†^.

Variable	Prophylactic Antibiotics Discontinued Within 24 h of Surgery	Prophylactic Antibiotics Continued for More than 24 h After Surgery	*p*-Value
	(N = 19,988, 24.13%)	(N = 62,852, 75.87%)	
Surgical site infections	31 (0.16%)	922 (1.47%)	<0.05
Pus or purulent drainage from incision sites or organs	8 (0.04%)	136 (0.22%)	<0.05
Positive culture results from incision sites or organs	1 (0.01%)	85 (0.14%)	<0.05
Surgical wounds that ruptured spontaneously or were opened by a surgeon, with one or more signs of infection	5 (0.03%)	120 (0.19%)	<0.05
Evidence of abscess or infection in the deep incision site or in organs or cavities in histopathological examination, radiological examination, etc.	0 (0.00%)	17 (0.03%)	<0.05
Diagnosis of surgical site infections by the surgeon, attending physician or infectious disease specialist	17 (0.09%)	620 (0.99%)	<0.05
Non-surgical-site infections	122 (0.61%)	2817 (4.48%)	<0.05
Total postoperative infections	153 (0.77%)	3652 (5.81%)	<0.05

^†^ Values are N (%).

**Table 3:** Multivariable logistic regression analysis for each outcome in patients who had either decompression, instrumented fusion, vertebroplasty, or kyphoplasty ^†^.

Variables ^††^	Surgical Site Infections ^a^	Non-Surgical-Site Infections ^b^	Total Postoperative Infections ^c^
Group			
≥24 h discontinuation group	10.732 (7.296–15.785) *	16.062 (13.114–19.671) *	17.823 (14.828–21.422) *
<24 h discontinuation group			
Age	1.008 (1.002–1.015) *	1.007 (1.003–1.010) *	1.007 (1.004–1.011) *
Sex, male	1.150 (1.002–1.320) *	0.924 (0.845–1.009)	0.982 (0.907–1.063)
QA period (sixth, seventh, eighth QA vs. ninth QA)	0.345 (0.297–0.401) *	0.056 (0.049–0.064) *	0.080 (0.072–0.088) *
Antibiotics used			
Other antibiotics only	1.555 (1.055–2.292) *	1.724 (1.355–2.195) *	1.764 (1.427–2.182) *
1st- or 2nd-generation cephalosporin, and other antibiotics—both	4.475 (3.877–5.165) *	5.928 (5.418–6.485) *	6.523 (6.011–7.079) *
1st- or 2nd-generation cephalosporin only	Reference	Reference	Reference
Insurance types (medical aids vs. health insurance)	0.807 (0.607–1.073)	1.121 (0.948–1.324)	1.029 (0.882–1.201)
Hospital types			
Tertiary	3.029 (2.502–3.666) *	5.658 (4.952–6.464) *	6.005 (5.318–6.780) *
Secondary	2.626 (2.239–3.080) *	1.858 (1.675–2.062) *	2.160 (1.967–2.372) *
Primary	Reference	Reference	Reference
Surgery type			
Decompression	3.795 (2.525–5.704) *	0.957 (0.789–1.160)	1.254 (1.049–1.500) *
Instrumented fusion	4.248 (2.734–6.601) *	0.827 (0.662–1.034)	1.122 (0.914–1.378)
Vertebroplasty and kyphoplasty	Reference	Reference	Reference
Comorbidity/medical history			
Diabetes mellitus	1.143 (0.989–1.321)	0.955 (0.871–1.048)	1.004 (0.923–1.091)
Hypertension	1.335 (1.150–1.549) *	1.339 (1.221–1.469) *	1.396 (1.285–1.518) *
Malnutrition	1.831 (1.329–2.521) *	1.260 (0.995–1.597)	1.551 (1.258–1.913) *
Skin or soft tissue infection	1.289 (0.996–1.667)	0.893 (0.745–1.071)	1.022 (0.870–1.201)
Allergy to antibiotics	1.177 (0.748–1.852)	0.934 (0.698–1.248)	0.953 (0.735–1.236)
Operation time (minutes) ^d^	1.271 (1.096–1.473) *	1.417 (1.295–1.549) *	1.408 (1.297–1.527) *
Number of concomitant spine surgeries	1.405 (1.245–1.586) *	1.377 (1.271–1.492) *	1.495 (1.390–1.608) *

^†^ Values are OR (95% CI), with asterisks (*) added for statistical significance. ^††^ The reference level is the latter (i.e., A vs. B means that B was the reference level). ^a^ Max-rescaled R^2^ = 0.216. ^b^ Max-rescaled R^2^ = 0.367. ^c^ Max-rescaled R^2^ = 0.382. ^d^ Calculated as a log value due to its exponential distribution by nature.

## Data Availability

The data presented in this study were obtained from the Health Insurance Review and Assessment Service (HIRA) and are subject to restricted access due to privacy regulations. Requests for data access may be directed to HIRA.

## Supplementary Materials

The following supporting information can be downloaded at https://www.mdpi.com/article/10.3390/antibiotics15030272/s1. Figure S1: Changes in the prevalence of each assessment item across sixth, seventh, eighth, and ninth QA. Table S1. Baseline characteristics of study participants who had either instrumented fusion or decompression. Table S2. Postoperative infection differences between the <24 h discontinuation group and ≥24 h discontinuation group in patients who had either instrumented fusion or decompression. Table S3. Multivariable logistic regression analysis for each outcome in patients who had either instrumented fusion or decompression. Table S4. Surgery site for patients categorized by surgery type.

## Abbreviations

The following abbreviations are used in this manuscript:

aOR	Adjusted Odds Ratio
ASHP	American Society of Health-System Pharmacists
BMI	Body Mass Index
CDC	Centers for Disease Control and Prevention
CI	Confidence Interval
HIRA	Health Insurance Review and Assessment Service
ICD-10	International Classification of Diseases, tenth Revision
IDSA	Infectious Diseases Society of America
QA	Quality Assessment
SSI	Surgical Site Infection
