# Supplementary material for: Early Discontinuation of Prophylactic Antibiotics Is Not Associated with Increased Surgical Site Infection Risk in Spine Surgery: A Nationwide Cohort Study
Source: Antibiotics (Basel). 2026 Mar 6;15(3):272. doi: 10.3390/antibiotics15030272 (PMC13023957; doi:10.3390/antibiotics15030272)
Supplement: Supplementary file 1 [file antibiotics-15-00272-s001.zip › antibiotics-4162892-supplementary.pdf]

Supplemental Materials

TITLE: Early Discontinuation of Prophylactic Antibiotics Is Not Associated with Increased Surgical Site Infection Risk in Spine Surgery: A Nationwide Cohort Study

CONTENTS

Figure S1. Changes in the prevalence of each assessment item across 6th, 7th, 8th, and 9th QA. 2

Table S1. Baseline characteristics of study participants who had either instrumented fusion or decompression. † 3

Table S2. Postoperative infection differences between the <24h discontinuation group and ≥24h discontinuation group in patients who had either instrumented fusion or decompression. † 5

Table S3. Multivariable logistic regression analysis for each outcome in patients who had either instrumented fusion or decompression. † 6

Table S4. Surgery site for patients categorized by surgery type........7

Figure S1. Changes in the prevalence of each assessment item across 6th, 7th, 8th, and 9th QA.

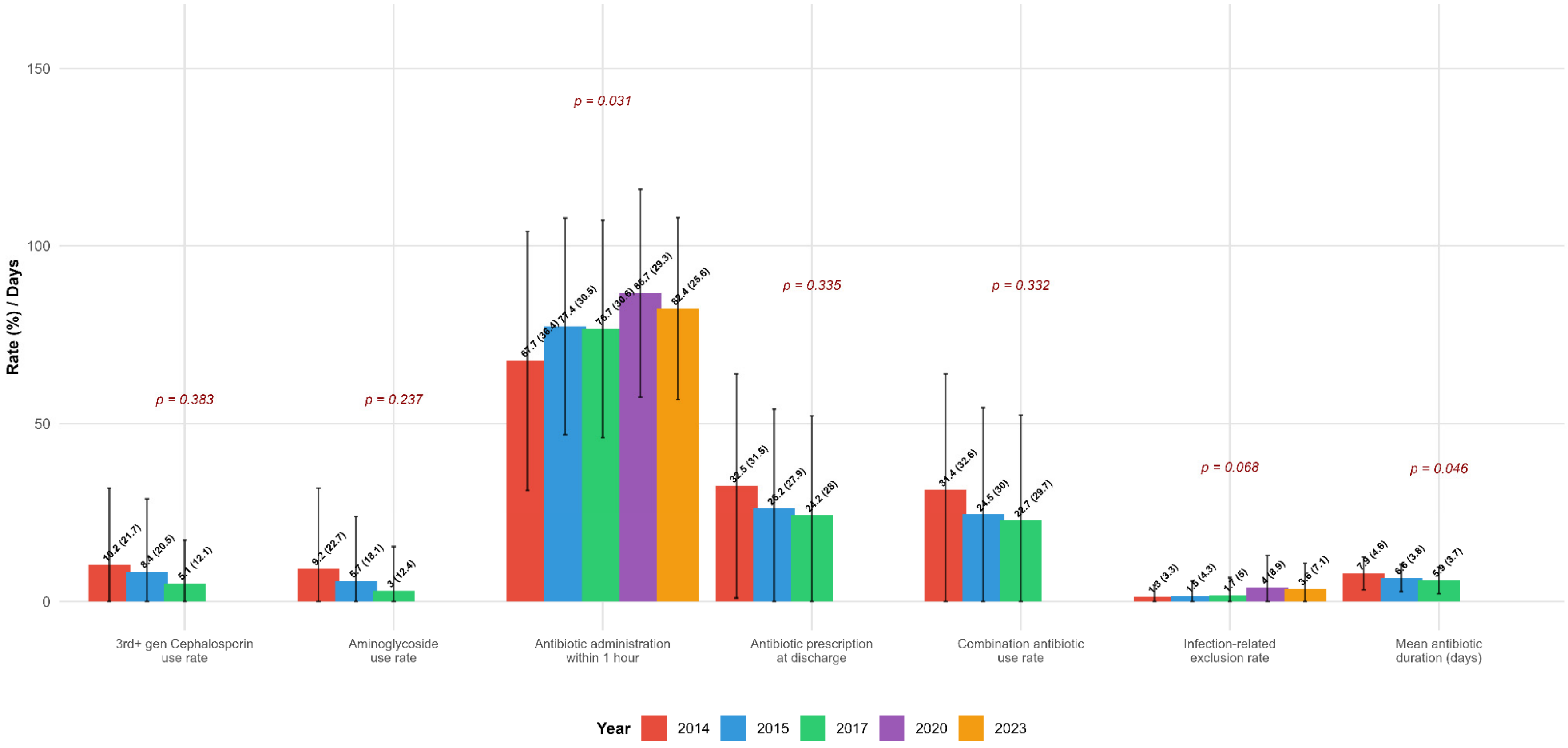

* The values are represented as mean (SD), with Δ as the difference between the surgical site infection group and non-surgical site infection group.

* Difference in means between the years was tested with analysis of variance (ANOVA).

Table S1. Baseline characteristics of study participants who had either instrumented fusion or decompression. †

Characteristics	Prophylactic antibiotics discontinued within 24 hours of surgery (N=9,754)	Prophylactic antibiotics continued for more than 24 hours after surgery (N=49,249)	P-value
Age (years), means (SD)	58.95 (14.64)	59.00 (13.96)	0.77
Round			<0.05
6th	134 (1.37%)	201 (0.41%)	
7th	896 (9.19%)	14,186 (28.80%)	
8th	1,203 (12.33%)	16,240 (32.98%)	
9th	7,521 (77.11%)	18,622 (37.81%)	
Sex			<0.05
Male	5,402 (55.38%)	26,488 (53.78%)	
Female	4,352 (44.62%)	22,761 (46.22%)	
Comorbidities [a]			
DM	2,675 (27.42%)	13,087 (26.57%)	0.08
Hypertension	3,593 (36.84%)	16,554 (33.61%)	<0.05
History [a]			
Malnutrition	196 (2.01%)	657 (1.33%)	<0.05
Uncontrolled DM	59 (0.60%)	239 (0.49%)	0.13
Skin or soft tissue infection	400 (4.10%)	2,036 (4.13%)	0.88
Insurance types			0.12
Health insurance coverage	9,341 (95.77%)	46,985 (95.40%)	
Medical aids	413 (4.23%)	2,264 (4.60%)	
Hospital types			<0.05
Tertiary	4,236 (43.43%)	4,483 (9.10%)	
General	2,418 (24.79%)	7,507 (15.24%)	
Hospital	3,100 (31.78%)	37,259 (75.65%)	
Antibiotics used			<0.05
1st or 2nd generation cephalosporin only	9,319 (95.54%)	39,927 (81.07%)	
Other antibiotics only	246 (2.52%)	1,810 (3.68%)	

1st or 2nd generation cephalosporin, and other antibiotics both	189 (1.94%)	7,512 (15.25%)	
Surgery site			<0.05
Cervical	1,736 (20.86%)	4,848 (10.26%)	
Thoracic	244 (2.93%)	494 (1.05%)	
Lumbar	6,342 (76.21%)	41,893 (88.69%)	
Surgery type			<0.05
Decompression	7,047 (72.25%)	38,925 (79.04%)	
Instrumented fusion	2,707 (27.75%)	10,324 (20.96%)	
Allergy to antibiotics, Presence	175 (1.79%)	993 (2.02%)	0.15
The number of concomitant spine surgeries, means (SD)	1.50 (0.67)	1.34 (0.56)	<0.05
Operation times (minutes)	100.0 (65.0-151.0)	100.0 (70.0-160.0)	0.07
Total hospitalization days (days)	8.0 (5.0-11.0)	11.0 (8.0-15.0)	<0.05

[†] Values are either mean (SD), median (IQR),

[a] Positive if there was a past history before discharge.

Table S2. Postoperative infection differences between the <24h discontinuation group and ≥24h discontinuation group in patients who had either instrumented fusion or decompression. †

Variable	Total	Prophylactic antibiotics discontinued within 24 hours of surgery	Prophylactic antibiotics continued for more than 24 hours after surgery	P value
	**(N=82,840)**	**(N=19,988)**	**(N=62,852)**	
Surgical site infections	953 (1.15%)	31 (0.16%)	922 (1.47%)	<0.05
Pus or Purulent drainage from incision sites or organs	144 (0.17%)	8 (0.04%)	136 (0.22%)	<0.05
Positive culture results from incision sites or organs	86 (0.10%)	1 (0.01%)	85 (0.14%)	<0.05
Surgical wounds that ruptured spontaneously or were opened by a surgeon, with one or more signs of infection	125 (0.15%)	5 (0.03%)	120 (0.19%)	<0.05
Evidence of abscess or infection in the deep incision site or in organs or cavities in histopathological examination, radiological examination, etc.	17 (0.02%)	0 (0.00%)	17 (0.03%)	<0.05
Diagnosis of surgical site infections by the surgeon, attending physician or infectious disease specialist	637 (0.77%)	17 (0.09%)	620 (0.99%)	<0.05
Non-surgical site infections	2,939 (3.55%)	122 (0.61%)	2,817 (4.48%)	<0.05
Total postoperative infections	3,805 (4.59%)	153 (0.77%)	3,652 (5.81%)	<0.05

† Values are N (%).

Table S3. Multivariable logistic regression analysis for each outcome in patients who had either instrumented fusion or decompression. †

**Variables** ††	**Surgical site infections** [a]	**Non-surgical site infections** [b]	**Total postoperative infections** [c]
Group			
≥24h discontinuation group	15.250 (9.538-24.383)*	31.439 (23.888-41.377)*	33.677 (26.439-42.896)*
<24h discontinuation group			
Age	1.008 (1.002-1.014)*	1.005 (1.001-1.009)*	1.006 (1.002-1.009)*
Sex, Male	1.140 (0.991-1.311)	0.895 (0.816-0.981)*	0.961 (0.884-1.044)
QA period (6th, 7th, 8th QA vs. 9th QA)	0.361 (0.310-0.420)*	0.048 (0.041-0.055)*	0.075 (0.067-0.084)*
Antibiotics used			
Other antibiotics only	1.555 (1.041-2.322)*	1.601 (1.221-2.098)*	1.671 (1.322-2.112)*
1st or 2nd generation cephalosporin, and other antibiotics both	4.514 (3.899-5.225)*	4.783 (4.344-5.266)*	5.611 (5.143-6.122)*
1st or 2nd generation cephalosporin only	Reference	Reference	Reference
Insurance types (Medical aids vs. Health insurance coverage)	0.762 (0.565-1.028)	1.062 (0.881-1.281)	0.960 (0.808-1.140)
Hospital types			
Tertiary	3.141 (2.591-3.809)*	6.026 (5.247-6.920)*	6.448 (5.686-7.314)*
Secondary	2.798 (2.378-3.293)*	1.831 (1.632-2.055)*	2.234 (2.018-2.473)*
Primary	Reference	Reference	Reference
Surgery type (Instrumented fusion vs. Decompression)	1.115 (0.946-1.313)	0.835 (0.746-0.935)*	0.865 (0.782-0.957)*
Comorbidity/Medical history			
Diabetes mellitus	1.167 (1.006-1.353)*	0.946 (0.855-1.045)	1.003 (0.917-1.097)
Hypertension	1.347 (1.156-1.569)*	1.404 (1.271-1.552)*	1.461 (1.336-1.597)*
Malnutrition	1.784 (1.275-2.498)*	1.084 (0.818-1.437)	1.430 (1.119-1.828)*
Skin or soft tissue infection	1.267 (0.971-1.653)	0.901 (0.740-1.097)	1.033 (0.868-1.229)
Allergy to antibiotics	1.174 (0.738-1.866)	0.989 (0.729-1.343)	0.999 (0.761-1.313)
Operation time (minutes) [d]	1.273 (1.094-1.482)*	1.662 (1.506-1.834)*	1.599 (1.464-1.747)*
The number of concomitant spine surgeries	1.412 (1.249-1.596)*	1.329 (1.223-1.444)*	1.464 (1.357-1.579)*

† Values are OR (95% CI), with asterisks (*) added for statistical significance.

†† The reference level is the latter (i.e. A vs. B means that B was the reference level.).

[a] Max-rescaled R-Square = 0.216

[b] Max-rescaled R-Square = 0.367

[c] Max-rescaled R-Square = 0.382

[d] Calculated as a log value due to its exponential distribution of nature.

Table S4. Surgery site for patients categorized by surgery type.

Surgery Site	Prophylactic antibiotics discontinued within 24 hours of surgery	Prophylactic antibiotics continued for more than 24 hours after surgery	P value
	(N=19,988)	(N=62,852)	
Patients who had either decompression, instrumental fusion, vertebroplasty, or kyphoplasty [a]			< 0.050
Cervical	1,736 (20.86%)	4,848 (10.26%)	
Thoracic	244 (2.93%)	494 (1.05%)	
Lumbar	6,342 (76.21%)	41,893 (88.69%)	
Patients who had decompression [b]			< 0.050
Cervical	675 (9.58%)	1,838 (4.72%)	
Thoracic	176 (2.50%)	386 (0.99%)	
Lumbar	4,764 (67.60%)	34,687 (89.11%)	
Patients who had instrumented fusion [c]			< 0.050
Cervical	1,061 (39.19%)	3,010 (29.16%)	
Thoracic	68 (2.51%)	108 (1.05%)	
Lumbar	1,578 (58.29%)	7,206 (69.80%)	

[a] Number of missing cases were 11,666 (58.37%) and 15,617 (24.85%) for <24h discontinuation group and ≥24h discontinuation group.

[b] Missing cases were 1,432 (20.32%) and 2,014 (5.17%) for <24h discontinuation group and ≥24h discontinuation group.

[c] There were no missing cases for patients who had instrumented fusion.
